# Stratified by baseline corticosteroid use: which advanced therapy works best for whom in ulcerative colitis?

**DOI:** 10.3389/fmed.2026.1938069

**Published:** 2026-09-29

**Authors:** Mengqing Dong, Zhicheng Li, Hongwei Hua, Qinghan Ma, Xingli Xu, Jiong Wu

**Affiliations:** Department of Proctology, Yueyang Hospital of Integrated Traditional Chinese and Western Medicine, Shanghai University of Traditional Chinese Medicine, Shanghai, China

**Keywords:** biologics, corticosteroids, efficacy, JAK inhibitors, stratified analysis, ulcerative colitis

## Abstract

**Background:**

Corticosteroids (CS) are commonly used as bridging therapy in ulcerative colitis (UC), but whether baseline CS use modifies the subsequent efficacy of advanced therapies remains unclear. With differing mechanisms across drug classes, it is uncertain whether CS impact on outcomes is consistent or drug-class-dependent.

**Methods:**

We searched PubMed, Embase, Web of Science, and Cochrane Library for CS-stratified randomized controlled trials (RCTs) comparing active drugs with placebo in moderate-to-severe UC. After deduplication, studies were selected per predefined criteria. Bias risk was assessed with the Cochrane tool. Pooled risk ratios (RRs) with 95% confidence intervals (CIs) were calculated using a random-effects model; robustness was tested by leave-one-out sensitivity analyses.

**Results:**

Ten RCTs (7 publications, 6,286 patients) were included. Overall, CS-naïve patients showed significantly higher rates of endoscopic improvement (RR = 1.91, 95% CI: 1.33–2.74, *p* < 0.001, I^2^ = 0%), symptom relief (RR = 1.63, 95% CI: 1.16–2.29, *p* = 0.005, I^2^ = 0%) and clinical remission (RR = 1.50, 95% CI: 1.03–2.19, *p* = 0.03, I^2^ = 0%) than CS users during induction; no significant differences were observed for other efficacy endpoints. This overall trend masked marked heterogeneity by drug class. IL-23 inhibitors exhibited a pronounced advantage in non-CS users across multiple endpoints, including clinical remission, clinical response, and endoscopic improvement. S1P modulators yielded the largest effect size for histologic–endoscopic mucosal improvement (RR = 3.04, 95% CI: 1.18–7.86, *p* = 0.02). No significant differences were found between groups in adverse events, serious adverse events, discontinuations, infections, acne, or CPK elevation, even with CS use up to 52 weeks.

**Conclusions:**

Baseline CS status may influence the efficacy of advanced therapies in UC. IL-23 inhibitors appeared to benefit CS-naïve patients across multiple endpoints, whereas JAK inhibitors showed a numerical but nonsignificant advantage in CS-exposed patients. Short-term concomitant CS did not significantly increase adverse events, supporting the clinical acceptability of CS bridging in highly active disease. These findings support stratified reporting in future RCTs and call for prospective studies to validate these observations.

**Systematic Review Registration:**

https://www.crd.york.ac.uk/PROSPERO/view/CRD420251248449, identifier, CRD420251248449.

## 1 Introduction

Ulcerative colitis (UC) is a chronic, immune-mediated inflammatory bowel disease (IBD) primarily affecting the colon and rectum (1). Its clinical course is characterized by relapsing symptoms including abdominal pain, diarrhea, and mucopurulent bloody stools (2). Corticosteroids (CS) are widely used to induce remission in active UC; however, while effective for symptom control, they often fail to achieve mucosal healing and are associated with considerable adverse effects (3, 4). Short-term complications include acne, moon face, and edema (5, 6), whereas long-term exposure increases the risk of cataracts, osteoporosis, and serious infections (3, 7).

Clinical guidelines recommend limiting CS use to a maximum of three months and advise prompt transition to steroid-free regimens in steroid-dependent or steroid-refractory patients (8). Despite these recommendations, CS overuse and long-term dependence remain prevalent, posing substantial health risks and economic burden (9). Approximately 30% of patients with IBD receive CS therapy, of whom 15%–20% develop overuse or dependence; this proportion is even higher among those with moderate-to-severe UC (10).

With the advent of advanced therapies—including biologics such as anti-integrin agents (e.g., vedolizumab) (11) and anti-interleukin agents (e.g., mirikizumab, risankizumab) (12), as well as small-molecule drugs such as JAK inhibitors (e.g., upadacitinib, tofacitinib) (13) and S1P receptor modulators (e.g., etrasimod) (14)—the management of moderate-to-severe UC has evolved substantially. These agents act through distinct mechanisms, modulating immune cell trafficking, cytokine signaling, or lymphocyte circulation to achieve more targeted inflammation control (15).

Despite guideline advocacy for early CS discontinuation to minimize long-term toxicity (16), clinicians frequently continue CS to prevent post-withdrawal relapse, creating uncertainty in clinical practice (17). Critically, pivotal trials of advanced therapies have largely reported overall efficacy estimates without stratification by baseline CS use, leaving an important question unanswered: does baseline CS status modify treatment response, and if so, does this effect differ across drug classes? Given the distinct and potentially complementary mechanisms of action among advanced therapies, the interaction between CS exposure and specific drug targets may be heterogeneous—a possibility that has not been systematically evaluated.

To address this evidence gap, we conducted a systematic review and meta-analysis of high-quality randomized controlled trials to determine whether baseline CS use modifies the efficacy and safety of advanced therapies in UC. Furthermore, by comparing CS-naïve and CS-exposed patients across different drug classes, we aimed to identify potential treatment-by-subgroup interactions and provide evidence-based guidance for individualized therapeutic decisions during the transition from CS to advanced therapy.

## 2 Materials and methods

### 2.1 Study registration

This study was prospectively registered in the PROSPERO database (CRD420251248449) and was conducted in accordance with the Preferred Reporting Items for Systematic Reviews and Meta-Analyses (PRISMA) guidelines (18). The completed PRISMA checklist is provided as Supplementary Material (Table S6).

### 2.2 Literature search and study selection

To identify relevant studies, we systematically searched PubMed, Embase, Web of Science, and the Cochrane Library from inception to December 1, 2025. The search strategy combined subject headings and free-text terms to comprehensively identify randomized controlled trials (RCTs) evaluating biologics and small-molecule therapies for IBD. The full search syntax for each database is provided in the Supplementary Material. Two reviewers independently screened titles and abstracts, and subsequently the full texts of retrieved articles, against the predefined eligibility criteria. Disagreements regarding study inclusion were resolved through discussion or, when necessary, by consultation with a third reviewer.

### 2.3 Inclusion criteria

We included phase II or III parallel-group randomized controlled trials that enrolled adults (≥18 years) with moderate-to-severe active ulcerative colitis (modified Mayo score 6–12, endoscopic subscore ≥2). Eligible trials had to report at least one efficacy or safety outcome stratified by baseline oral corticosteroid use. The intervention was required to be an approved advanced therapy for ulcerative colitis, administered for a minimum of 14 days, and the control had to be placebo.

### 2.4 Exclusion criteria

We excluded studies that enrolled patients with acute severe ulcerative colitis, evaluated non-oral corticosteroid therapies, did not stratify patients by baseline oral corticosteroid use, or did not report efficacy or safety outcomes stratified by corticosteroid subgroup.

### 2.5 Outcome definition

The outcome definitions listed below were extracted from the protocols and primary publications of the included trials and are presented here to specify the criteria for data extraction and pooling. A cross-trial verification is provided in Supplementary Tables S1 and S2. The primary outcomes during the induction phase were clinical remission (Adapted Mayo score: daily average stool frequency subscore ≤1 based on average daily stool frequency over the past 3 days and not higher than baseline, rectal bleeding subscore = 0, and endoscopic subscore ≤1 without friability), clinical response (≥2-point and ≥30% decrease in Adapted Mayo score from baseline, with a decrease in rectal bleeding subscore ≥1 or an absolute subscore ≤1), endoscopic improvement (endoscopic subscore ≤1 without friability), symptom relief (complete absence of bowel urgency, abdominal pain, nocturnal bowel movements, tenesmus, and fecal incontinence, along with improvements in fatigue and sleep disruption. Failure to achieve complete absence of any of these listed symptoms (or the required improvements) was considered as lack of symptom relief), endoscopic remission (endoscopic subscore = 0), and histologic–endoscopic mucosal improvement (HEMI; endoscopic subscore 0 or 1 without friability and Geboes score ≤3.1).

Secondary outcomes assessed during the induction phase included treatment-emergent adverse events (TEAEs), serious adverse events (SAEs), adverse events leading to discontinuation, infections, acne, and creatine phosphokinase elevation. Outcomes evaluated during the maintenance phase comprised clinical remission, clinical response, endoscopic remission, TEAEs, SAEs, discontinuations due to adverse events and infections.

### 2.6 Data extraction and quality assessment

Two reviewers independently extracted data using a standardized form, which captured study characteristics (first author, publication year, country, setting, sample size, efficacy and safety outcomes), patient demographics, dosage of the active drug, corticosteroid use, and outcome measures according to the definitions described in Section 2.5. Discrepancies were resolved by re-examining the original articles or through consultation with a third reviewer.

The risk of bias in each included RCT was assessed using the Cochrane Collaboration's revised Risk of Bias 2 (RoB 2) tool (19). Two independent reviewers evaluated each trial across six predefined domains: the randomization process, deviations from intended interventions, missing outcome data, measurement of the outcome, selection of the reported result, and overall risk of bias. Each domain was judged as low risk, some concerns, or high risk. Disagreements between the two reviewers were resolved by consulting a third reviewer. To ensure consistency and harmonization of judgments, all assessors underwent calibration exercises using the RoB 2 tool prior to the formal assessment.

### 2.7 Statistical analysis

We pooled dichotomous outcomes using risk ratios (RRs) with 95% confidence intervals (CIs) under a random-effects model (DerSimonian-Laird method) to account for anticipated clinical and methodological heterogeneity. To evaluate effect modification by CS use, interaction RRs were calculated within each trial (active drug vs. placebo) comparing CS-naïve with CS-exposed patients. Subgroup standard errors were derived from reported 95% CIs, and log-transformed interaction estimates were pooled using the generic inverse-variance method under a random-effects model. An interaction RR > 1 indicated greater efficacy in CS-naïve patients, and <1 in CS-exposed patients; *p* < 0.05 was considered statistically significant.

Heterogeneity was assessed via the Cochran Q test and quantified by the I^2^ statistic, defined as negligible (<30%), low (30%–49%), moderate (50%–74%), or high (>75%) (20). We performed leave-one-out sensitivity analyses to assess the impact of individual studies on pooled results. Given the limited number of included studies, formal publication-bias assessment was not undertaken (21). Subgroup analyses explored CS-associated effects across drug classes. Since no included trial directly compared advanced therapies, cross-drug comparisons relied on class-specific pooled estimates and represent indirect comparisons of effect modification, not direct head-to-head comparisons. All analyses were performed using Review Manager 5.4 (Cochrane Collaboration).

## 3 Results

### 3.1 Literature search

A total of 4,732 references were identified through database searches. After removing 2023 duplicates, 2,709 unique references were screened according to the inclusion criteria. According to the study design, 2,512 records were excluded due to non-RCT study design. Following the screening of titles and abstracts, 37 records were excluded because their interventions were not advanced therapies. Of the remaining 160 studies, 153 were excluded. Among these exclusions, 130 lacked outcome data stratified by CS, 12 enrolled patients with acute critical illness during hospitalization, 2 were animal experiments, 9 focused on pediatric populations, and Trials of phase II and TNF antagonists were excluded due to the lack of extractable corticosteroid-stratified outcome data. Consequently, 7 reports (22–28) were assessed for eligibility, leading to the final inclusion of studies in the review. The search methods and study selection process were conducted in accordance with the PRISMA statement, as illustrated in Figure 1. Detailed information on the included studies is provided in Tables 1, 2.

**Figure 1:**
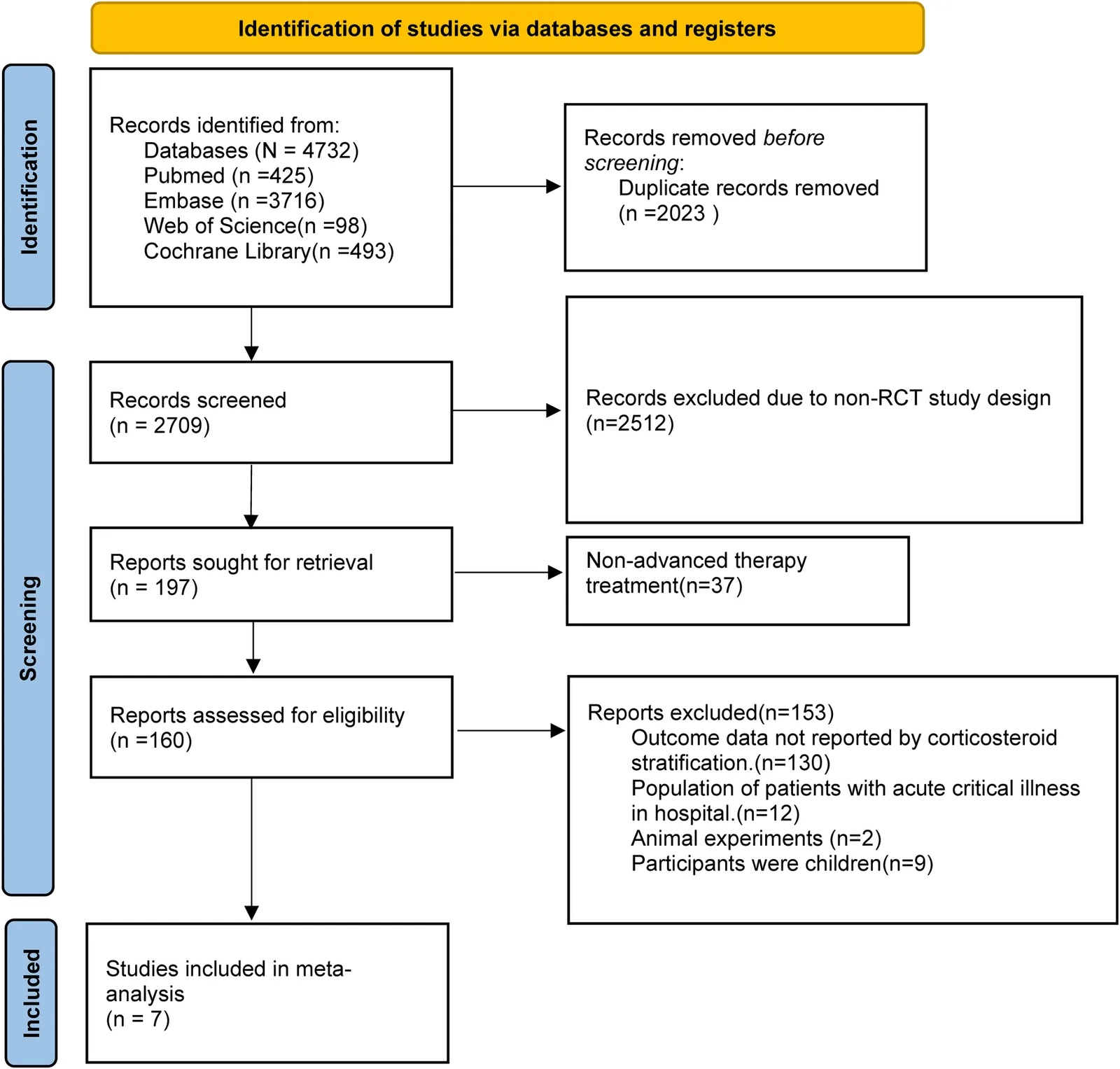
PRISMA flow chart for the systematic search and selection process.

**Table 1:** Characteristic of included studies during the induction phase.

Ref.	Setting	Intervention	Control	Indication	Age	Male, *n* (%)	CS use among CS-treated patients	Patients (CS use)
Wu et al. (22), 2025, China	Randomized, double-blind, placebo-controlled, multicenter, phase 3 study	Oral Etrasimod 2 mg, QD	Placebo	Moderately to severely active ulcerative colitis	41.7	208 (61.2%)	Oral, Induction period dose remained stable	Etrasimod: 11 Placebo: 6
Sands et al. (23), 2025, United States	Randomized, double-blind, placebo-controlled, multicenter, phase 3 study	Oral Etrasimod 2 mg, QD	Placebo	Moderately to severely active ulcerative colitis	ELEVATE UC 12: 35–41 ELEVATE UC 52: 39–41	NA	Oral prednisone, budesonide, or equivalent; Mean dose: ∼15 mg/day (prednisone equivalent); Stable during induction (first 12 weeks)	ELEVATE UC 12: Etrasimod: 65 Placebo: 34 ELEVATE UC 52: Etrasimod: 93 Placebo: 42
Feagan et al. (24), 2013, Canada	Randomized, double-blind, placebo-controlled, multicenter, phase 3 study	Vedolizumab IV 300 mg, weeks 0 and 2	Placebo	Moderately to severely active ulcerative colitis	40	525 (58.7%)	Oral prednisone (or equivalent); Median dose: 20 mg/day (IQR 10–30 mg); Stable during induction (until week 6)	Vedolizumab:79 Placebo:58
D’Haens et al. (25), 2023, Netherlands	Randomized, double-blind, placebo-controlled, multicenter, phase 3 study	Mirikizumab 300 mg IV, Q4W	Placebo	Moderately to severely active ulcerative colitis	42	695 (59.8%)	Oral,Induction period dose remained stable	Mirikizumab: 351 Placebo: 113
Sandborn et al. (26), 2017, United States	Randomized, double-blind, placebo-controlled, multicenter, phase 3 study	Oral Tofacitinib 10 mg, BID	Placebo	Moderately to severely active ulcerative colitis	40–43	567 (49.8%)	Oral glucocorticoids (prednisone or equivalent); Maximum dose allowed: 25 mg/day; Dose during induction: Stable during induction (until week 8)	OCTAVE Induction 1: Tofacitinib: 214 Placebo: 58 OCTAVE Induction 2: Tofacitinib: 198 Placebo: 55
Danese et al. (27), 2022, Italy	Randomized, double-blind, placebo-controlled, multicenter, phase 3 study	Oral Upadactinib 45 mg, QD	Placebo	Moderately to severely active ulcerative colitis	UC1: Upadactinib: 42 Placebo: 41 UC2: Upadactinib: 42 Placebo: 40	616 (62.3%)	Oral corticosteroid (prednisone equivalent); Median dose: 20 mg/day (range 15–30 mg); Stable during induction phase	UC1: Upadactinib: 128 Placebo: 63 UC2: Upadactinib: 146 Placebo: 72
Reinisch et al. (28), 2025, Austria	Randomized, double-blind, placebo-controlled, multicenter, phase 3 study	Risankizumab 1,200 mg IV, weeks 0, 4 and 8	Placebo	Moderately to severely active ulcerative colitis	42.1	586 (60.1%)	Oral prednisone (or equivalent); Median dose: 20 mg/day (IQR 10–20 mg); Stable during induction	Risankizumab: 236 Placebo: 112

**Table 2:** Characteristic of included studies during the maintenance phase.

Ref.	Maintenance (weeks)	Research design	Intervention	Control	CS use among CS-treated patients	Patients (CS use)
Wu et al. (22), 2025, China	40	1:1 re-randomization to etrasimod 2 mg/placebo at maintenance	Oral Etrasimod 2 mg, QD	Placebo	Oral, mandatory tapering	Etrasimod: 4 Placebo: 2
Sands et al. (23), 2025, United States	40	all-comers maintenance extension	Oral Etrasimod 2 mg, QD	Placebo	Oral, standardized CS tapering protocol (Prednisone: >10→5 mg/wk to 10 mg, then 2.5 mg/wk; ≤10→2.5 mg/wk; Budesonide 9 mg: EOD→E3D)	ELEVATE UC 52: Etrasimod: 93 Placebo: 42
Feagan et al. (24), 2013, Canada	46	1:1:1 re-randomization to vedolizumab every 8 weeks/every 4 weeks/placebo at maintenance	Vedolizumab IV 300 mg, every 4 or 8 weeks	Placebo	Oral, protocol-defined CS tapering	Vedolizumab: 143 (q8w group: 70; q4w group: 73) Placebo:72
D’Haens et al. (25), 2023, Netherlands	40	2:1 re-randomization to mirikizumab 200 mg every 4 weeks/placebo at maintenance	Mirikizumab 300 mg IV, Q4W	Placebo	Oral, protocol-defined CS tapering	Mirikizumab: 135 Placebo: 68
Sandborn et al. (26), 2017, United States	52	1:1:1 re-randomization to tofacitinib 5 mg twice daily/tofacitinib 10 mg twice daily/placebo for maintenance	Oral Tofacitinib 5 mg or 10 mg, BID	Placebo	Oral, protocol-defined CS tapering	NA
Danese et al. (27), 2022, Italy	52	1:1:1 re-randomization to Upadacitinib 15 mg once daily/Upadacitinib 30 mg once daily/placebo for maintenance	Oral Upadactinib 15 mg or 30 mg, QD	Placebo	Oral, Prednisone >10 mg/day: −5 mg/week; ≤10 mg/day: −2–5 mg/week; Budesonide: −3 mg/week; Beclomethasone: discontinue	UC3: Upadactinib: 139 (15 mg group: 53, 30 mg group: 86) Placebo: 85
Reinisch et al. (28), 2025, Austria	52	1:1:1 re-randomization to Risankizumab 180 mg SC/Risankizumab 360 mg SC/placebo (withdrawal) every 8 weeks for maintenance	Risankizumab 180 mg/360 mg SC, Q8W	Placebo	Oral, Prednisone (>10 mg/d): ↓5 mg/wk → 10 mg, then ↓2.5 mg/wk; Prednisone (≤10 mg/d): ↓2.5 mg/wk; Budesonide (≤9 mg/d): ↓3 mg/wk Bude-MX/Beclo: Stop	NA

### 3.2 Study characteristics

Baseline characteristics during the induction phase are summarized in Table 1. The mean age of participants ranged from 40 to 45 years. The proportion of male participants ranged from 49.8% to 62.3% across the included trials. The ten included trials were multicenter, phase 3 randomized, double-blind, placebo-controlled clinical trials. All studies used placebo for control arms, while the intervention arms consisted of Etrasimod, Upadacitinib, Vedolizumab, Mirikizumab, Tofacitinib, and Risankizumab. The patients had moderate to severe ulcerative colitis. And the corticosteroid exposure and the number of corticosteroid-treated patients during the induction phase are detailed in the Table 1.

Baseline characteristics during the maintenance phase are summarized in Table 2. The maintenance phase duration varied from 40 to 52 weeks. The research designs were primarily categorized as re-randomization and all-comers maintenance extension. The intervention drugs and control measures during the maintenance phase were consistent with those in the induction phase. And the corticosteroid exposure and the number of corticosteroid-treated patients during the maintenance phase are detailed in the Table 2.

### 3.3 Risk of bias of the included studies

The results of the risk of bias assessment are presented in Figure 2. Overall, two studies were rated as having some concerns, while the remaining six studies showed a low risk of bias across all domains.

**Figure 2:**
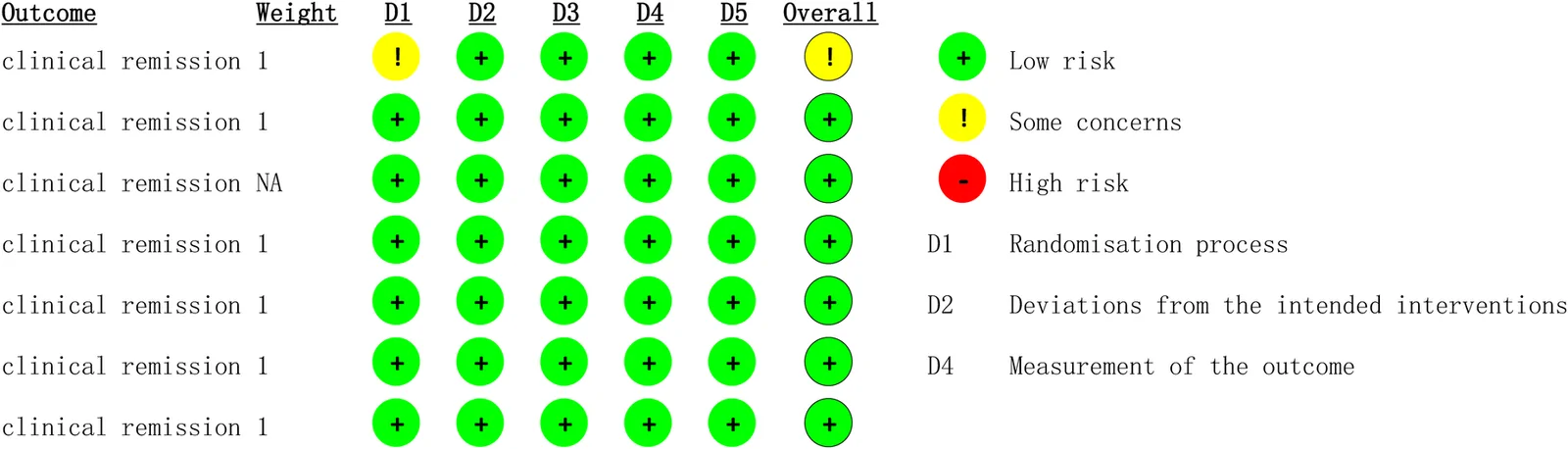
Summary and traffic light plots of the risk of bias of the included randomized controlled trials (22–28).

### 3.4 Primary outcomes

#### 3.4.1 Overall effect of baseline corticosteroid use

When all advanced therapies were considered together, CS-naïve patients demonstrated significantly higher rates of endoscopic improvement (RR = 1.91, 95% CI: 1.33–2.74, *p* < 0.001) (Figure 3), symptom relief (RR = 1.63, 95% CI: 1.16–2.29, *p* = 0.005) (Figure 4), and clinical remission (RR = 1.50, 95% CI: 1.03–2.19, *p* = 0.03) (Figure 6) during induction compared to CS-exposed patients. No significant overall differences were observed for clinical response (RR = 1.33, 95% CI: 0.99–1.78, *p* = 0.06) (Figure 5), endoscopic remission (RR = 1.46, 95% CI: 0.72–2.94, *p* = 0.29) (Figure 7), or histologic-endoscopic mucosal improvement (HEMI) (RR = 1.62, 95% CI: 0.99–2.63, *p* = 0.05) (Figure 8).

**Figure 3:**
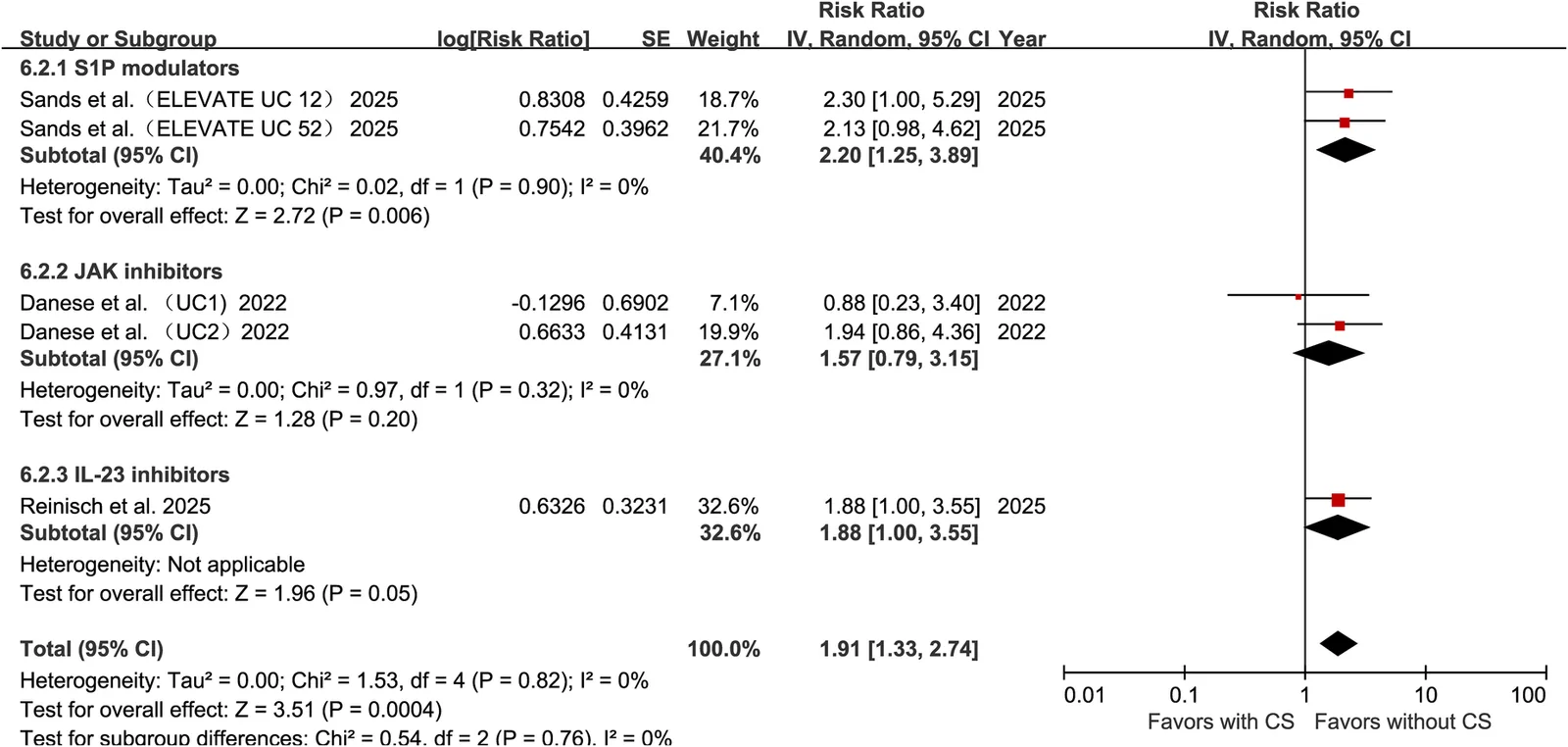
Forest plot of the incidence of endoscopic improvement during the induction phase.

**Figure 4:**
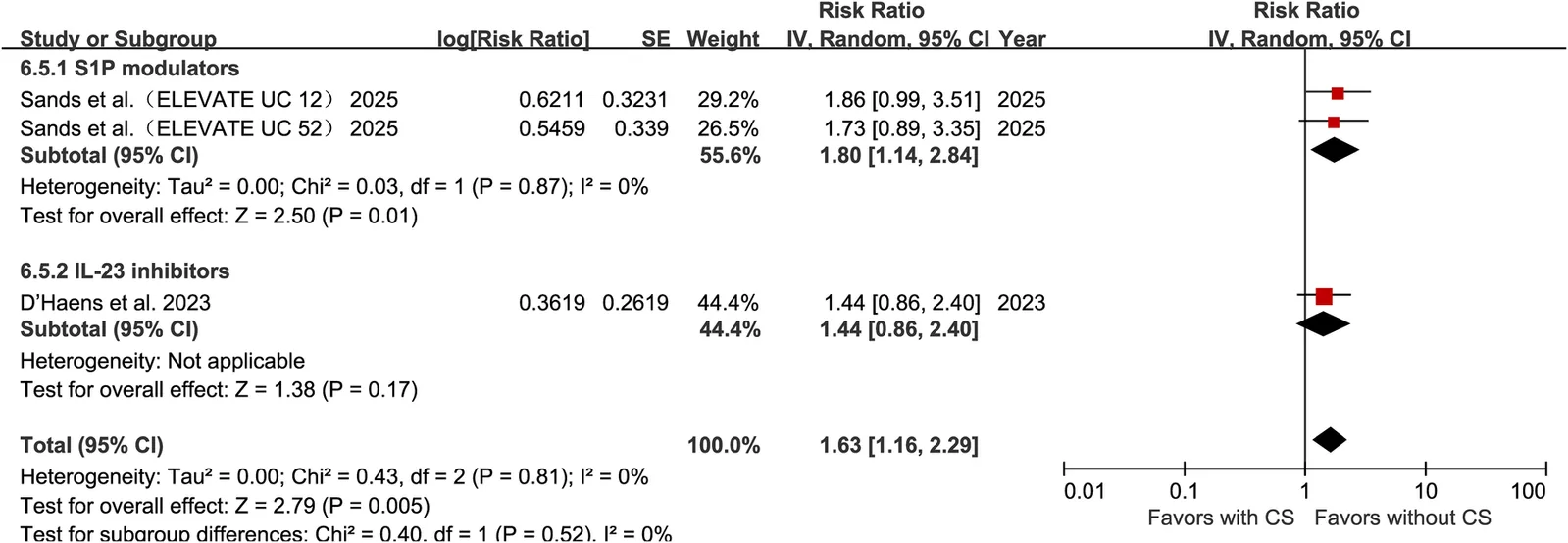
Forest plot of the incidence of symptom relief during the induction phase.

**Figure 5:**
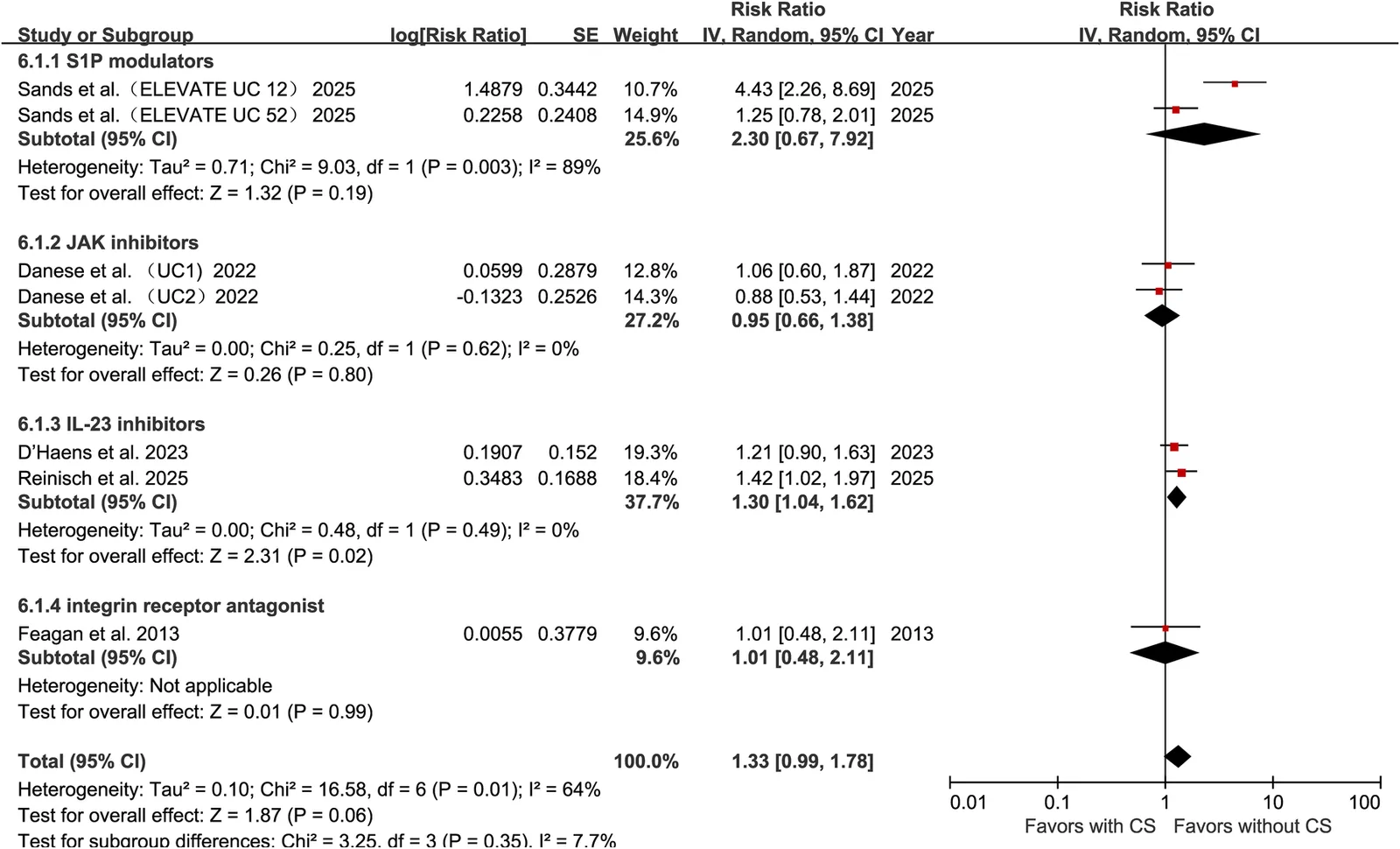
Forest plot of the incidence of clinical response during the induction phase.

**Figure 6:**
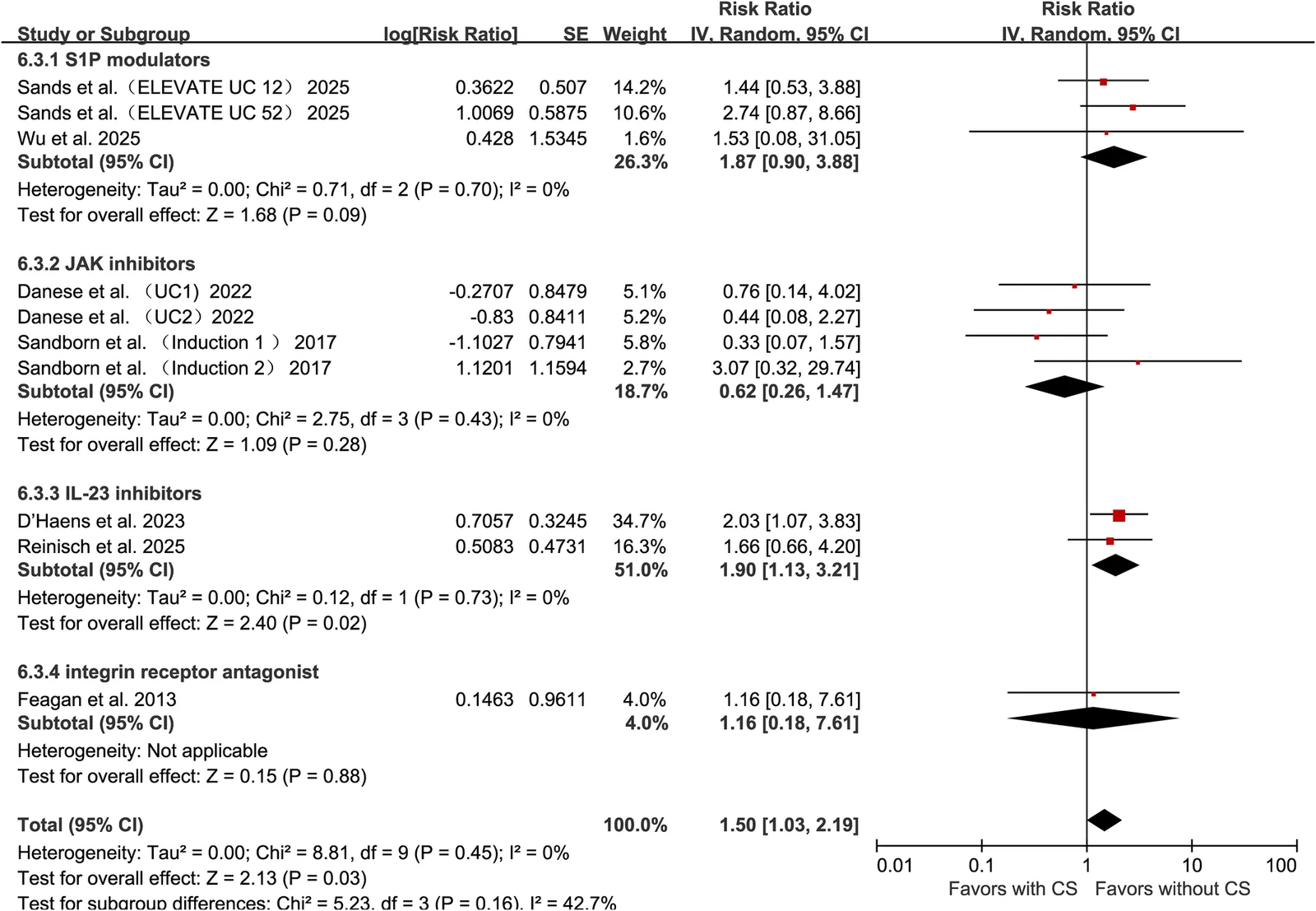
Forest plot of the incidence of clinical remission during the induction phase.

**Figure 7:**
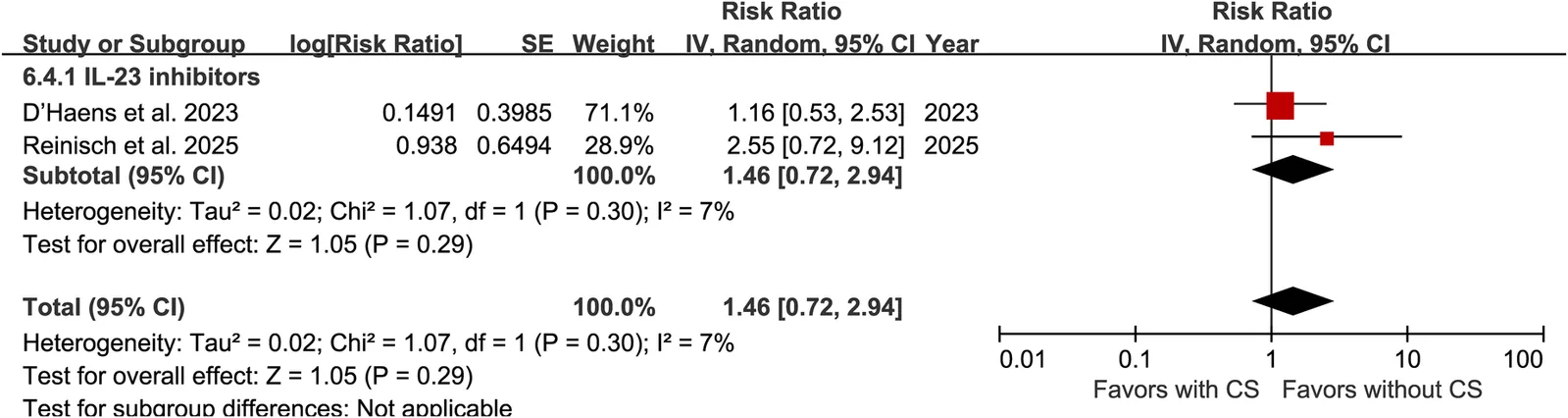
Forest plot of the incidence of endoscopic remission during the induction phase.

**Figure 8:**
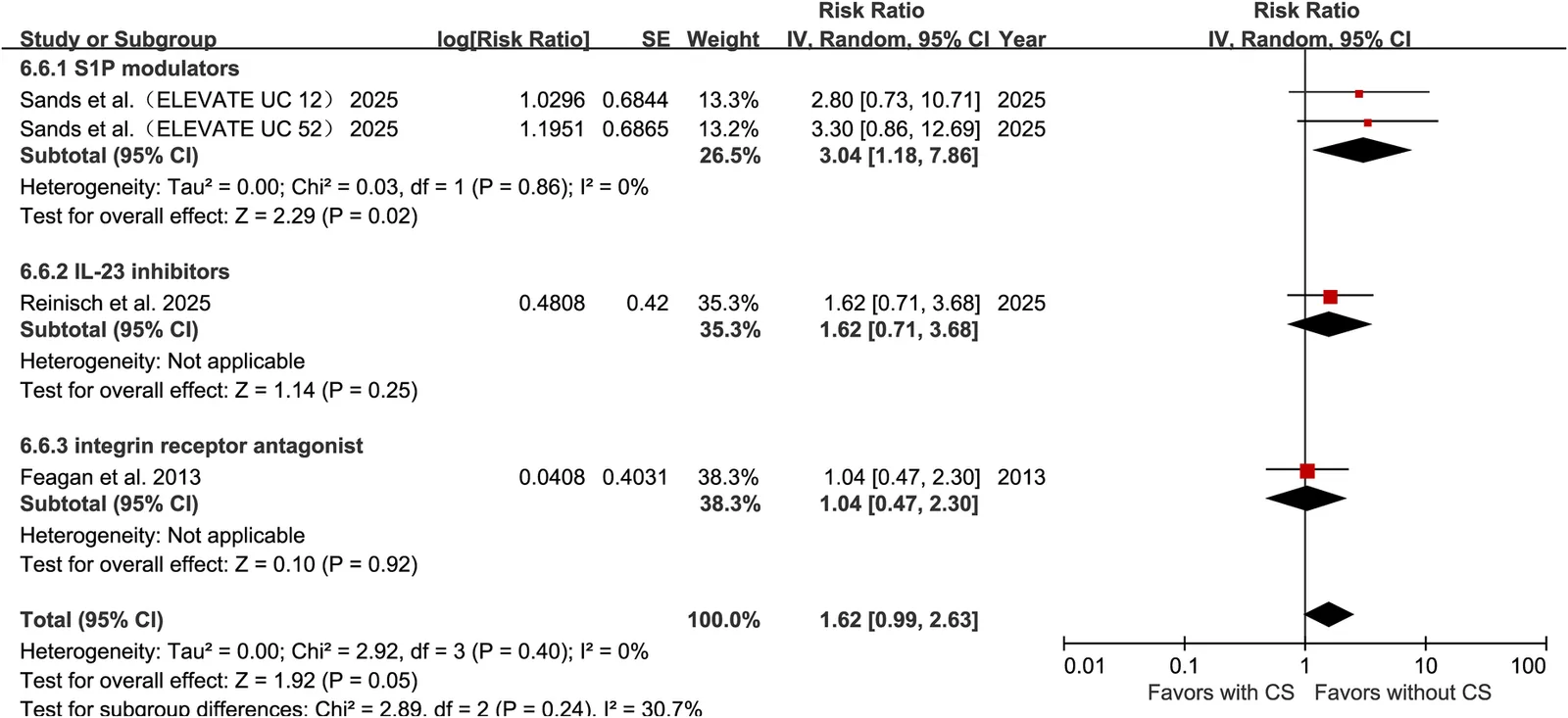
Forest plot of the incidence of histologic-endoscopic mucosal improvement during the induction phase.

#### 3.4.2 Subgroup analysis by drug class

To explore whether the impact of baseline CS use was consistent across different mechanisms of action, we performed subgroup analyses stratified by drug class. Formal tests for interaction did not reveal statistically significant differences among drug classes for any of the efficacy endpoints (*p* for interaction ranged from 0.16 to 0.76), indicating that the modifying effect of CS status was not definitively drug-class-dependent. However, several descriptive patterns emerged (Figures 3–8).

For IL-23 inhibitors, patients without baseline CS use consistently showed numerically better outcomes across multiple endpoints, including endoscopic improvement (RR = 1.88, 95% CI: 1.00–3.55) (Figure 3), clinical response (RR = 1.30, 95% CI: 1.04–1.62) (Figure 5), and clinical remission (RR = 1.90, 95% CI: 1.13–3.21) (Figure 6).

In patients treated with S1P modulators, a pronounced benefit for CS-naïve patients was observed for the composite endpoint of HEMI (RR = 3.04, 95% CI: 1.18–7.86) (Figure 8), as well as for endoscopic improvement (RR = 2.20, 95% CI: 1.25–3.89) (Figure 3) and symptom relief (RR = 1.80, 95% CI: 1.14–2.84) (Figure 4).

In contrast to the above patterns, JAK inhibitors showed a numerical but non-significant trend favoring CS-exposed patients for clinical remission (RR = 0.62, 95% CI: 0.26–1.47) (Figure 6); no significant CS-related differences were found for other endpoints with this class.

For integrin receptor antagonists, only a single trial of vedolizumab provided limited data, showing no significant CS-related differences for any outcome assessed.

Collectively, these descriptive patterns highlight potential class-specific trends that warrant cautious interpretation and prospective validation.

### 3.5 Secondary outcomes

CS use during the induction period did not significantly increase the risk of adverse outcomes. Specifically, regardless of baseline CS use, no statistically significant differences were observed in the incidence of TEAEs (RR = 1.02, 95% CI: 0.87–1.20, *p* = 0.78, I^2^ = 0%) (Supplementary Figure S1), SAEs (RR = 1.24, 95% CI: 0.61–2.51, *p* = 0.56, I^2^ = 0%) (Supplementary Figure S2), or adverse events leading to discontinuation (RR = 1.05, 95% CI: 0.33–3.36, *p* = 0.94, I^2^ = 0%) (Supplementary Figure S3). For specific adverse events, similarly, no significant differences were observed for specific adverse events, including acne (RR = 1.29, 95% CI: 0.17–9.95, *p* = 0.80, I^2^ = 0%) (Supplementary Figure S4), creatine phosphokinase elevation (RR = 1.01, 95% CI: 0.15–6.95, *p* = 0.99, I^2^ = 0%) (Supplementary Figure S5) or infections (RR = 0.41, 95% CI: 0.05–3.10, *p* = 0.39, I^2^ = 0%) (Supplementary Figure S6).

During the maintenance phase, the presence or absence of baseline CS did not significantly influence any efficacy outcome, including clinical remission (RR = 0.89, 95% CI: 0.59–1.34, *p* = 0.57, I^2^ = 0%) (Supplementary Figure S7), clinical response (RR = 1.05, 95% CI: 0.78–1.42, *p* = 0.75, I^2^ = 4%) (Supplementary Figure S8), or endoscopic remission (RR = 1.20, 95% CI: 0.39–3.66, *p* = 0.75, I^2^ = 72%) (Supplementary Figure S9). Safety outcomes were similarly unaffected, with no significant differences observed in TEAEs (RR = 1.00, 95% CI: 0.90–1.11, *p* = 0.98, I^2^ = 0%) (Supplementary Figure S10), SAEs (RR = 1.56, 95% CI: 0.73–3.34, *p* = 0.25, I^2^ = 44%) (Supplementary Figure S11), adverse events leading to discontinuation (RR = 1.84, 95% CI: 0.27–12.61, *p* = 0.53, I^2^ = 12%) (Supplementary Figure S12) or infections (RR = 1.08, 95% CI: 0.57–2.05, *p* = 0.81, I^2^ = 0%) (Supplementary Figure S13).

This study found no evidence that CS use during the induction and maintenance phases (up to 52 weeks) significantly increases the risk of adverse events. However, their long-term safety still warrants further investigation.

### 3.6 Heterogeneity

To investigate potential sources of the high heterogeneity observed in the clinical response and clinical remission outcomes, we performed leave-one-out sensitivity analyses. For clinical response, the primary analysis including all studies showed no significant difference and moderate heterogeneity (RR = 1.33, 95% CI: 0.99–1.78, *p* = 0.06, I^2^ = 64%) (Figure 5). After excluding the Sands et al. (ELEVATE UC 12) study, the pooled estimate became statistically significant and heterogeneity was eliminated (RR = 1.19, 95% CI: 1.01–1.42, *p* = 0.04, I^2^ = 0%) (Supplementary Figure S14). This heterogeneity was primarily attributable to the ELEVATE UC 12 trial, in which steroid tapering was not permitted during the 12-week induction period; consequently, the CS subgroup had an unusually high placebo response rate, driven by sustained corticosteroid exposure that blunted the observed treatment effect.

## 4 Discussion

This study systematically assessed whether the impact of baseline CS use on the efficacy and safety of advanced therapies in UC varies by drug class. The overall analysis showed that CS-naïve patients achieved significantly higher rates of endoscopic improvement, symptom relief, and clinical remission during induction. IL-23 inhibitors showed numerically better outcomes in CS-naïve patients across multiple endpoints, S1P modulators demonstrated a pronounced effect on HEMI in CS-naïve patients, and JAK inhibitors exhibited a numerical but non-significant opposite trend favoring CS-exposed patients for clinical remission. Although no statistically significant differences were found among the drugs across subgroups for any outcome measure, the observed effect trends merit further exploration in future prospective studies.

To our knowledge, this is the first meta-analysis to systematically evaluate whether baseline CS use differentially modifies the efficacy of different drug classes. Previous meta-analyses have consistently demonstrated the overall efficacy of these agents in moderate-to-severe UC. While one recent meta-analysis performed corticosteroid-stratified analyses restricted to S1P-receptor modulators (29), no study to date has specifically evaluated the differential modification of treatment effects by baseline CS status across different drug classes (30–33). The present study fills this gap by providing stratified estimates derived from CS-prespecified subgroups within each trial, offering a more nuanced evidence base for clinical decision-making during the transition from CS to advanced therapy.

Several observations merit further discussion. First, the finding that S1P modulators yielded the largest effect size for HEMI in CS-naïve patients is noteworthy. HEMI is a composite endpoint reflecting both endoscopic and histologic improvement and is widely acknowledged to predict long-term disease control and relapse risk more reliably than symptom improvement alone. The pronounced effect on this deep remission endpoint suggests that S1P modulators may be particularly effective in achieving sustained mucosal healing when initiated in a steroid-free setting. Second, IL-23 inhibitors demonstrated consistent numerical advantages in CS-naïve patients across three hard endpoints: endoscopic improvement, clinical response, and clinical remission, without a corresponding signal in the more subjective symptom relief endpoint. This pattern may reflect that IL-23 inhibitors exert more pronounced effects on objective inflammatory endpoints (endoscopic improvement and clinical response) than on subjective symptom relief, though this remains speculative. Third, the numerical but non-significant trend favoring CS-exposed patients for JAK inhibitors in clinical remission (RR = 0.62, 95% CI: 0.26–1.47) directionally differed from the other drug classes and warrants prospective investigation in head-to-head trials.

CS are commonly used as bridging therapy to achieve rapid symptom control while awaiting response to advanced therapies (8, 16). Our safety data, showing no significant increase in adverse events with short-term concomitant CS use, lend support to the clinical acceptability of this bridging strategy in highly active disease. However, the class-specific efficacy patterns suggest that the choice of advanced therapy may need to consider the patient's CS status at treatment initiation. For patients who are CS-naïve, IL-23 inhibitors or S1P modulators may offer particular advantages in achieving deep remission endpoints, although these observations are descriptive and require confirmation in dedicated prospective studies. For patients who are already CS-exposed, the data are less clear, though JAK inhibitors showed a numerical trend that merits further study.

Several considerations should be taken into account when interpreting these findings. First, the number of studies with CS-stratified data was modest (seven publications, ten trials), and the CS subgroups were relatively small in some cases. Additional data from future RCTs would be valuable. Second, safety data were primarily derived from short-term follow-up (induction and up to 52 weeks of maintenance). Longer-term observation would further strengthen the evidence. Third, there were potential differences in patient populations across trials, including geographic regions (e.g., East Asia, North America, and Europe), prior biologic exposure and CS tapering protocols, which may have played a role in the observed heterogeneity. Notably, CS use was a prespecified stratification factor in most trials, which supports the comparability of treatment effects within each CS subgroup despite the above considerations.

## 5 Conclusion

This systematic review and meta-analysis demonstrates that baseline CS status may influence the efficacy of different advanced therapies in UC, with descriptive trends potentially favoring IL-23 inhibitors in CS-naïve patients**,** and JAK inhibitors in CS-exposed patients. Short-term concomitant CS did not significantly increase adverse events, supporting the clinical acceptability of CS bridging in highly active disease. Prospective studies with prespecified CS-stratified analyses are warranted to validate these descriptive observations.

## Data Availability

The original contributions presented in the study are included in the article/Supplementary Material, further inquiries can be directed to the corresponding author.
