# Supplementary material for: Stratified by baseline corticosteroid use: which advanced therapy works best for whom in ulcerative colitis?
Source: Front Med (Lausanne). 2026 Sep 29;13:1938069. doi: 10.3389/fmed.2026.1938069 (PMC13658562; doi:10.3389/fmed.2026.1938069)
Supplement: Supplementary file 1 [file Table1.docx]

# 1 **Supplementary Tables**

**Table S1.** Results of the analysis for all outcomes.

| Outcomes | Studies | RR | 95%CI | P value | Heterogeneity | GRADE |
| --- | --- | --- | --- | --- | --- | --- |
| **Induction phase** | | | | | | |
| endoscopic improvement | 5 | 1.91 | 1.33-2.74 | P<0.001 | 0% | High |
| symptom relief | 3 | 1.63 | 1.16-2.29 | P=0.005 | 0% | High |
| clinical response | 7 | 1.33 | 0.99-1.78 | P=0.06 | 64% | Moderate |
| clinical remission | 10 | 1.50 | 1.03-2.19 | P=0.03 | 0% | Moderate |
| endoscopic remission | 2 | 1.46 | 0.72-2.94 | P=0.29 | 7% | Moderate |
| histologic-endoscopic mucosal improvement | 4 | 1.62 | 0.99-2.63 | P=0.05 | 0% | High |
| treatment-emergent adverse events | 5 | 1.02 | 0.87-1.20 | P=0.78 | 0% | High |
| serious adverse events | 5 | 1.24 | 0.61-2.51 | P=0.56 | 0% | High |
| adverse events leading to discontinuation | 2 | 1.05 | 0.33-3.36 | P=0.94 | 0% | Moderate |
| acne | 2 | 1.29 | 0.17-9.95 | P=0.80 | 0% | Moderate |
| creatine phosphokinase elevation | 2 | 1.01 | 0.15-6.95 | P=0.99 | 0% | Moderate |
| infections | 3 | 0.41 | 0.05-3.10 | P=0.39 | 0% | High |
| **maintenance phase** | | | | | | |
| clinical remission | 5 | 0.89 | 0.59-1.34 | P=0.57 | 0% | High |
| clinical response | 3 | 1.05 | 0.78-1.42 | P=0.75 | 4% | High |
| endoscopic remission | 2 | 1.20 | 0.39-3.66 | P=0.75 | 72% | Moderate |
| treatment-emergent adverse events | 5 | 1.00 | 0.90-1.11 | P=0.98 | 0% | High |
| serious adverse events | 5 | 1.56 | 0.73-3.34 | P=0.25 | 44% | Moderate |
| adverse events leading to discontinuation | 2 | 1.84 | 0.27-12.61 | P=0.53 | 12% | Moderate |
| infections | 3 | 1.08 | 0.57-2.05 | P=0.81 | 0% | High |

**Table S2.** Efficacy outcomes of the studies during the induction phase.

| Ref. | Total patients | | Clinical remission | Clinical response | Endoscopic improvement | Endoscopic remission | Symptom relief | Histologicen-doscopic mucosal improvement |
| --- | --- | --- | --- | --- | --- | --- | --- | --- |
| Wu et al^[22]^, 2025, China | With CS Etrasimod: 11 Placebo: 6 | 2 0 | | NA | NA | NA | NA | NA |
|  | Without CS  Etrasimod: 217  Placebo: 106 | 55  6 | | NA | NA | NA | NA | NA |
| Sands et al^[23]^, 2025, United States | With CS  ELEVATE UC 12  Etrasimod: 65 Placebo: 34 ELEVATE UC 52  Etrasimod: 93 Placebo: 42 | 19 7  30 7 | | 38 18  63 19 | 22 11  38 12 | NA | 29 14  42 13 | 10 5  20 5 |
|  | Without CS ELEVATE UC 12 Etrasimod: 173 Placebo: 82 ELEVATE UC 52 Etrasimod: 196 Placebo: 102 | 43  10  51  5 | | 113  11  119  33 | 56  11  70  12 | NA | 85  20  92  19 | 31  5  46  4 |
| Feagan et al^[24]^, 2013, Canada | With CS Vedolizumab: 79  Placebo: 58 | 14 3 | | 37 15 | NA | NA | NA | 32 14 |
|  | Without CS Vedolizumab: 71  Placebo: 47 | 12  2 | | 33  12 | NA | NA | NA | 29  11 |
| D’Haens et al^[25]^, 2023, Netherlands | With CS Mirikizumab: 351 Placebo: 113 | 71 19 | | 206 48 | NA | 58 10 | 99 24 | NA |
|  | Without CS: Mirikizumab: 517 Placebo: 181 | 139  20 | | 338  71 | NA | 118  19 | 180  33 | NA |
| Sandborn et al^[26]^, 2017, United States | With CS OCTAVE Induction 1 Tofacitinib: 214 Placebo: 58 OCTAVE Induction 2 Tofacitinib: 198 Placebo: 55 | 36 2   33 3 | | NA | NA | NA | NA | NA |
|  | Without CS OCTAVE Induction 1 Tofacitinib: 262 Placebo: 64 OCTAVE Induction 2 Tofacitinib: 231 Placebo: 57 | 53  8  38  1 | | NA  NA | NA  NA | NA  NA | NA  NA | NA  NA |
| Danese et al^[27]^, 2022, Italy | With CS  UC1  Upadactinib: 124 Placebo: 53 UC2 Upadactinib: 118 Placebo: 103 | 30 2  34 2 | | 91  15  83  25 | 41  3  83  25 | NA | NA | NA |
|  | Without CS UC1 Upadactinib: 123 Placebo: 94 UC2 Upadactinib: 205 Placebo: 85 | 32  5  78  5 | | 90  25  153  25 | 47  7  95  7 | NA  NA | NA  NA | NA  NA |
| Reinisch et al^[28]^, 2025, Austria | With CS Risankizumab: 236 Placebo: 112 | 40 8 | | 129  43 | 73  17 | 18  5 | NA | 49  10 |
|  | Without CS Risankizumab: 414 Placebo: 213 | 92  12 | | 289  73 | 164  22 | 51  6 | NA | 110  15 |

**Table S3.** Safety outcomes of the studies during the induction phase.

| Ref. | Total patients | treatment-emergent adverse events (TEAEs) | serious adverse events (SAEs) | adverse events leading to discontinuation | acne | creatine phosphokinase elevation | infections |
| --- | --- | --- | --- | --- | --- | --- | --- |
| Feagan et al^[24]^, 2013, Canada | With CS Vedolizumab:  Placebo: 8 | 35  27 | 3  4 | NA | NA | NA | 1  1 |
|  | Without CS Vedolizumab: 1  Placebo: 7 | 32  22 | 2  3 | NA | NA | 0  1 | NA |
| D’Haens et al^[25]^, 2023, Netherlands | With CS Mirikizumab: 351 Placebo: 113 | 159  54 | 11  7 | 6  10 | NA | NA | 2  0 |
|  | Without CS Mirikizumab: 517 Placebo: 181 | 227  81 | 14  9 | 8  11 | NA | NA | 3  1 |
| Danese et al^[27]^, 2022, Italy | With CS UC1 Upadactinib: 124 Placebo: 53 UC2 Upadactinib: 118 Placebo: 103 | 67  31  60  43 | 3  3  3  4 | NA  NA | NA  NA | 6  0  10  2 | NA  NA |
|  | Without CS UC1 Upadactinib: 123 Placebo: 94 UC2  Upadactinib: 205 Placebo: 85 | 72  57  112  27 | 5  6  8  4 | NA  NA | 9  1  14  1 | 10  2  9  1 | NA  NA |
| Reinisch et al^[28]^, 2025, Austria | With CS Risankizumab: 236 Placebo: 112 | 112  58 | 4  11 | 2  4 | NA | NA | 2  1 |
|  | Without CS Risankizumab: 415 Placebo: 212 | 162  103 | 11  22 | 2  8 | NA | NA | 2  3 |

**Table S4.** Efficacy outcomes of the studies during the maintenance phase.

| Ref. | Total patients | Clinical remission | Clinical response | Endoscopic remission |
| --- | --- | --- | --- | --- |
| Wu et al^[22]^, 2025, China | With CS Etrasimod: 4 Placebo: 2 | 2 0 | NA | NA |
|  | Without CS Etrasimod: 73 Placebo: 78 | 35  10 | NA | NA |
| Sands et al^[23]^, 2025, United States | With CS ELEVATE UC 52 Etrasimod: 93 Placebo: 42 | 29  4 | 43  13 | 24  7 |
|  | Without CS ELEVATE UC 52 Etrasimod: 196 Placebo: 102 | 65  7 | 100  22 | 55  8 |
|  | Without CS Upadacitinib (15mg + 30mg): 290 Upadacitinib 15mg: 141  Upadacitinib 30mg: 149 Placebo: 139 | 140  61  79  17 | NA | NA |
| Feagan et al^[24]^, 2013, Canada | With CS Vedolizumab (Q4W+Q8W): 143  Vedolizumab Q4W: 73  Vedolizumab Q8W: 70  Placebo：72 | 62  33  29  11 | 77  38  39  17 | NA |
|  | Without CS Vedolizumab (Q4W+Q8W): 104  Vedolizumab Q4W: 52  Vedolizumab Q8W: 52  Placebo: 54 | 45  23  22  9 | 56  27  29  13 | NA |
| D’Haens et al^[25]^, 2023, Netherlands | With CS Mirikizumab: 135 Placebo: 68 | 61  12 | 98  27 | 66  13 |
|  | Without CS: Mirikizumab: 230 Placebo: 111 | 111  33 | 175  50 | 124  32 |
| Sandborn et al^[26]^, 2017, United States | With CS  Tofacitinib: NA Placebo: NA | NA | NA | NA |
|  | Without CS Tofacitinib: NA Placebo: NA | NA | NA | NA |
| Danese et al^[27]^, 2022, Italy | With CS UC3 Upadactinib (15mg + 30mg): 139  Upadactinib 15mg: 53  Upadactinib 30mg: 86 Placebo: 85 | 56  18  38  9 | NA | NA |
|  | Without CS UC3 Upadactinib (15mg + 30mg): 163  Upadactinib 15mg: 95  Upadactinib 30mg: 68 Placebo: 85 | 82  44  38  9 | NA | NA |
| Reinisch et al^[28]^, 2025, Austria | With CS Risankizumab: NA Placebo: NA | NA | NA | NA |
|  | Without CS Risankizumab: NA Placebo: NA | NA | NA | NA |

**Table S5.** Safety outcomes of the studies during the maintenance phase.

| Ref. | Total patients | treatment-emergent adverse events (TEAEs) | serious adverse events (SAEs) | adverse events leading to discontinuation | herpes zoster | infections |
| --- | --- | --- | --- | --- | --- | --- |
| Sands et al^[23]^, 2025, United States | With CS ELEVATE UC 52 Etrasimod: 158 Placebo: 76 | 94  39 | 7  8 | NA | 1  1 | 30  14 |
|  | Without CS ELEVATE UC 52 Etrasimod: 369 Placebo: 184 | 224  96 | 19  3 | NA | 1  1 | 73  38 |
|  | Without CS Upadacitinib (15mg + 30mg): 290 Upadacitinib 15mg: 141  Upadacitinib 30mg: 149 Placebo: 139 | NA | NA | NA | 17  6  11  0 | 13  7  6  5 |
| Feagan et al^[24]^, 2013, Canada | With CS Vedolizumab (Q4W+Q8W): 143  Vedolizumab Q4W: 73  Vedolizumab Q8W: 70  Placebo: 72 | 116  59  57  60 | 13  7  6  11 | NA | NA | 2  1  1  2 |
|  | Without CS Vedolizumab (Q4W+Q8W): 104  Vedolizumab Q4W: 52  Vedolizumab Q8W: 52  Placebo: 54 | 85  42  43  45 | 9  5  4  9 | NA | NA | 2  1  1  2 |
| D’Haens et al^[25]^, 2023, Netherlands | With CS Mirikizumab: 135 Placebo: 68 | 88  48 | 5  6 | 2  6 | NA | 2  0 |
|  | Without CS Mirikizumab: 230 Placebo: 111 | 147  75 | 147  75 | 3  8 | NA | 3  1 |
| Danese et al^[27]^, 2022, Italy | With CS UC3 Upadactinib (15mg + 30mg): 112  Upadactinib 15mg: 55  Upadactinib 30mg: 57 Placebo: 60 | 87  43  44  48 | 6  3  3  8 | NA | 6  3  3  0 | NA |
|  | Without CS UC3 Upadactinib (15mg + 30mg): 190  Upadactinib 15mg: 93  Upadactinib 30mg: 97 Placebo: 89 | 149  72  77  65 | 13  7  6  11 | NA | NA | NA |
| Reinisch et al^[28]^, 2025, Austria | With CS  Risankizumab (180mg +360mg): 142 Risankizumab 180mg: 80  Risankizumab 360mg: 62 Placebo: 78 | 107  58  49  58 | 9  4  5  6 | 4  2  2  0 | NA | NA |
|  | Without CS Risankizumab (180mg +360mg): 246 Risankizumab 180mg: 113  Risankizumab 360mg: 133 Placebo: 118 | 171  82  89  92 | 11  6  5  10 | 4  1  4  3 | 1  0  1  2 | NA |

**Supplementary Table S6.** Definitions of Efficacy Endpoints and Cross-Trial Consistency Assessment

| Efficacy endpoint | Core definition adopted in this meta-analysis | Uniform across trials? |
| --- | --- | --- |
|  |  |  |
| Clinical remission | Adapted Mayo score: stool frequency subscore ≤1 and not higher than baseline, rectal bleeding subscore = 0, and endoscopic subscore ≤1 without friability | Yes |
| Clinical response | ≥2‑point and ≥30% decrease in Adapted Mayo score from baseline, with a decrease in rectal bleeding subscore ≥1 or an absolute subscore ≤1 | Yes |
| Endoscopic improvement | Endoscopic subscore ≤1 without friability | Yes |
| Endoscopic remission | Endoscopic subscore = 0 | Yes |
| Histologic‑endoscopic mucosal improvement (HEMI) | Endoscopic subscore 0 or 1 without friability and Geboes score ≤3.1 | Yes |
| Symptom relief | Complete absence of bowel urgency, abdominal pain, nocturnal bowel movements, and fecal incontinence, along with improvements in fatigue and sleep disruption | No (minor variations) |

**Supplementary Table S7.** Definitions of Safety Endpoints and Cross-Trial Consistency Assessment

| Safety endpoint | Definition standard | Uniform across trials? |
| --- | --- | --- |
| TEAEs | Adverse events occurring after first dose of study drug, coded per MedDRA | Yes |
| SAEs | Adverse events meeting seriousness criteria per ICH guidelines, coded per MedDRA | Yes |
| Adverse events leading to discontinuation | Adverse events resulting in permanent withdrawal of study drug | Yes |
| Infections | Infectious adverse events coded per MedDRA | Yes |
| Acne | Acne‑related adverse events coded per MedDRA | Yes |
| Creatine phosphokinase (CPK) elevation | Elevation above upper limit of normal (ULN) per laboratory criteria | Yes |

**Table S8.** Search strategy.

| Database |  | Keywords | Results |
| --- | --- | --- | --- |
| PubMed | #1 | (Inflammatory Bowel Disease OR colitis, ulcerative [MeSH] OR ulcerative colitis OR (ulcerative AND colitis) | 158,024 |
|  | #2 | (Biologics OR biological products [MeSH] OR small molecules*[MeSH] OR “induction"[All Fields] OR “maintenance"[All Fields] OR anti-TNF OR " tumor necrosis factor inhibitors "[All Fields] OR infliximab OR adalimumab OR golimumab OR anti-integrin OR vedolizumab OR etrolizumab OR interleukin 23 antagonist [MeSH] OR ustekinumab OR mirikizumab OR guselkumab) OR (risankizumab) OR (JAK inhibitor [MeSH] OR tofacitinib OR filgotinib OR upadacitinib OR sphingosine-1-phosphate receptor modulator OR ozanimod OR etrasimod) | 9,228,778 |
|  | #3 | (Adrenal Cortex Hormones [MeSH] OR Hormones, Adrenal Cortex OR Corticoid OR Adrenal Cortex Hormone OR Cortex Hormone, Adrenal OR Hormone, Adrenal Cortex OR Corticosteroid OR Corticoids OR Corticosteroids) | 411,489 |
|  | #4 | Randomized Controlled Trial | 4260 |
|  |  | #1 AND #2 AND #3 AND #4 | 425 |
| Embase | #1 | 'ulcerative colitis'/exp OR 'ulcerative colitis':ti,ab,kw OR ((ulcerative NEAR/3 colitis):ti,ab,kw) | 132,251 |
|  | #2 | 'biological product'/exp OR 'tumor necrosis factor inhibitor'/exp OR 'interleukin 23'/exp OR 'janus kinase inhibitor'/exp OR 'sphingosine 1 phosphate receptor modulator'/exp OR biologics:ti,ab,kw OR 'small molecule*':ti,ab,kw OR infliximab:ti,ab,kw OR adalimumab:ti,ab,kw OR golimumab:ti,ab,kw OR vedolizumab:ti,ab,kw OR ustekinumab:ti,ab,kw OR tofacitinib:ti,ab,kw OR upadacitinib:ti,ab,kw OR ozanimod:ti,ab,kw OR etrasimod:ti,ab,kw OR filgotinib:ti,ab,kw | 1,446,956 |
|  | #3 | 'corticosteroid'/exp OR 'glucocorticoid'/exp OR 'methylprednisolone'/exp OR 'prednisolone'/exp OR corticosteroid*:ti,ab,kw OR glucocorticoid*:ti,ab,kw OR steroid*:ti,ab,kw OR prednisone:ti,ab,kw OR prednisolone:ti,ab,kw OR methylprednisolone:ti,ab,kw OR hydrocortisone:ti,ab,kw OR budesonide:ti,ab,kw | 1,721,292 |
|  | #4 | 'randomized controlled trial'/exp OR 'controlled clinical trial'/exp OR 'clinical trial'/exp OR random*:ti,ab,kw OR trial:ti OR 'clinical study':ti | 4,200,128 |
|  |  | #1 AND #2 AND #3 AND #4 | 3,716 |
| Web of Science | #1 | ("Inflammatory Bowel Disease" OR "ulcerative colitis") NOT ("Crohn* disease" OR "CD " OR "IBD-unclassif*") | 122,329 |
|  | #2 | biologic* OR "small molecule*" OR "anti-TNF" OR "TNF inhibitor*" OR infliximab OR adalimumab OR golimumab OR certolizumab OR "anti-integrin" OR vedolizumab OR etrolizumab OR "interleukin 23 antagonist*" OR ustekinumab OR mirikizumab OR guselkumab OR risankizumab OR "JAK inhibitor*" OR tofacitinib OR filgotinib OR upadacitinib OR "sphingosine-1-phosphate receptor modulator*" OR ozanimod OR etrasimod | 2,161,403 |
|  | #3 | "Adrenal Cortex Hormone*" OR Corticosteroid* OR Corticoid* OR steroid* OR glucocorticoid* OR prednisone OR budesonide OR methylprednisolone | 623,226 |
|  | #4 | "randomized controlled trial" OR "randomised controlled trial" OR RCT | 320,655 |
|  |  | #1 AND #2 AND #3 AND #4 | 98 |
| Cochrane Library | #1 | (Inflammatory Bowel Disease) OR (colitis, ulcerative) OR (ulcerative colitis) OR (ulcerative AND colitis) | 11,032 |
|  | #2 | (Biologics) OR (biological products) OR (small molecules) OR (induction) OR (maintenance) OR (anti‐TNF) OR (tumor necrosis factor inhibitors) OR (infliximab) OR (adalimumab) OR (golimumab) OR (anti‐integrin) OR (vedolizumab) OR (etrolizumab) OR (interleukin 23 antagonist) OR (ustekinumab) OR (mirikizumab) OR (guselkumab) OR (risankizumab) OR (JAK inhibitor) OR (tofacitinib) OR (filgotinib) OR (upadacitinib) OR (sphingosine‐1‐phosphate receptor modulator) OR (ozanimod) OR (etrasimod) | 177,088 |
|  | #3 | (Adrenal Cortex Hormones) OR (Corticoid) OR (Adrenal Cortex Hormone) OR (Cortex Hormone, Adrenal) OR (Corticosteroid) OR (Corticoids) OR (Corticosteroids) | 29,372 |
|  | #4 | "randomized controlled trial" | 747,186 |
|  |  | #1 AND #2 AND #3 AND #4 | 493 |

# 2 **Supplementary Figures**

# **2.1 Forest plots for the induction phase**

**Figure S1.** Forest plot of treatment-emergent adverse events during the induction phase.

**Figure S2.** Forest plot of serious adverse events during the induction phase.

**Figure S3.** Forest plot of adverse events leading to discontinuation during the induction phase.

**Figure S4.** Forest plot of acne during the induction phase.

**Figure S5.** Forest plot of creatine phosphokinase elevation during the induction phase.

**Figure S6.** Forest plot of infections during the induction phase.

# **2.2 Forest plots for the maintenance phase (with CS vs without CS)**

**Figure S7.** Forest plot of the incidence of clinical remission during the maintenance phase.

**Figure S8.** Forest plot of the incidence of clinical response during the maintenance phase.

**Figure S9.** Forest plot of the incidence of endoscopic remission during the maintenance phase.

**Figure S10.** Forest plot of the incidence of treatment-emergent adverse events (TEAEs) during the maintenance phase.

**Figure S11.** Forest plot of the incidence of serious adverse events (SAEs) during the maintenance phase.

**Figure S12.** Forest plot of the incidence of adverse events leading to discontinuation during the maintenance phase.

**Figure S13.** Forest plot of the incidence of infections during the maintenance phase.

# **2.3 Sensitivity analysis**

**Figure S14.** Leave-one-out sensitivity analysis for clinical response after excluding the Sands et al. (ELEVATE UC 12) study.
